# (+)-Chlorido[(1,2,3,4-η;κ*P* ^2′^)-2′-diphenyl­phosphanyl-2-diphenyl­phosphoryl-1,1′-binaphth­yl]rhodium(I) methanol monosolvate

**DOI:** 10.1107/S1600536812003418

**Published:** 2012-02-10

**Authors:** Antje Meissner, Carmen Selle, Hans-Joachim Drexler, Detlef Heller

**Affiliations:** a Leibniz-Institut für Katalyse e.V. an der Universität Rostock, Albert-Einstein-Strasse 29a, 18059 Rostock, Germany

## Abstract

In the title complex, [RhCl(C_44_H_32_OP_2_)]·CH_3_OH, the Rh^I^ ion is coordinated by a naphthyl group of a partially oxidized 2,2′-bis­(diphenyl­phosphan­yl)-1,1′-binaphthyl (BINAP) ligand in a η^4^ mode, one P atom of the diphenyl­phosphanyl group and one Cl atom. The P=O group does not inter­act with the Rh^I^ ion but accepts an O—H⋯O hydrogen bond from the methanol solvent mol­ecule.

## Related literature

For general synthetic aspects of related compounds, see: Bunten *et al.* (2002 ▶). For related structures of rhodium complexes with BINAP and bis­phosphine diolefin, see: Fischer *et al.* (2012 ▶); Preetz (2009 ▶); Preetz *et al.* (2010 ▶); Tani *et al.* (1985 ▶).
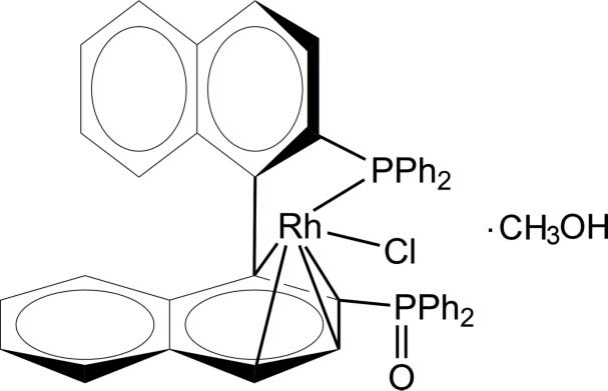


## Experimental

### 

#### Crystal data

[RhCl(C_44_H_32_OP_2_)]·CH_4_O
*M* *_r_* = 809.04
Triclinic, 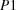

*a* = 9.2108 (18) Å
*b* = 9.7453 (19) Å
*c* = 11.354 (2) Å
α = 103.01 (3)°
β = 104.59 (3)°
γ = 105.09 (3)°
*V* = 905.0 (4) Å^3^
*Z* = 1
Mo *K*α radiation
μ = 0.67 mm^−1^
*T* = 200 K
0.20 × 0.15 × 0.15 mm

#### Data collection

Stoe IPDS-2 diffractometer
Absorption correction: numerical (*X-SHAPE* and *X-RED*; Stoe & Cie, 2002 ▶) *T*_min_ = 0.787, *T*_max_ = 0.953
10887 measured reflections
6247 independent reflections
5695 reflections with *I* > 2σ(*I*)
*R*_int_ = 0.025

#### Refinement

*R*[*F* ^2^ > 2σ(*F* ^2^)] = 0.027
*wR*(*F* ^2^) = 0.043
*S* = 0.96
6247 reflections
462 parameters
3 restraints
H-atom parameters constrained
Δρ_max_ = 0.64 e Å^−3^
Δρ_min_ = −0.34 e Å^−3^
Absolute structure: Flack (1983 ▶), 2858 Friedel pairs
Flack parameter: −0.012 (17)

### 

Data collection: *X-AREA* (Stoe & Cie, 2002 ▶); cell refinement: *X-AREA*; data reduction: *X-RED* (Stoe & Cie, 2002 ▶); program(s) used to solve structure: *SHELXS97* (Sheldrick, 2008 ▶); program(s) used to refine structure: *SHELXL97* (Sheldrick, 2008 ▶); molecular graphics: *SHELXTL* (Sheldrick, 2008 ▶); software used to prepare material for publication: *SHELXTL*.

## Supplementary Material

Crystal structure: contains datablock(s) I, global. DOI: 10.1107/S1600536812003418/hy2508sup1.cif

Structure factors: contains datablock(s) I. DOI: 10.1107/S1600536812003418/hy2508Isup2.hkl

Additional supplementary materials:  crystallographic information; 3D view; checkCIF report

## Figures and Tables

**Table 1:** Hydrogen-bond geometry (Å, °)

*D*—H⋯*A*	*D*—H	H⋯*A*	*D*⋯*A*	*D*—H⋯*A*
O51—H51⋯O1^i^	0.82	1.95	2.755 (4)	169

Symmetry code: (i) 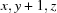.

**Fig. 1.**
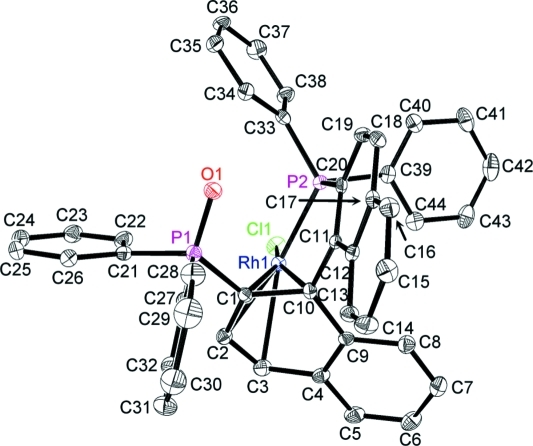
Molecular structure of the title compound. H atoms and the solvent molecule MeOH have been omitted for clarity. Displacement ellipsoids are drawn at the 30% probability level.

## supplementary crystallographic information

### Comment

Ligand exchange between 1,5-cyclooctadiene (COD) and 2,2'-bis(diphenylphosphanyl)-1,1'-binaphthyl (BINAP, a chiral binaphthyl-based chelating diphosphine) in the complex [Rh(COD)(µ_2_-Cl)]_2_ has been investigated by Preetz (2009) and Bunten *et al.* (2002). When the reaction is carried out at room temperature, either in dichloromethane or toluene, the complex [Rh(BINAP)(µ_2_-Cl)]_2_ is formed in 99% yield. The title complex, [Rh(BINAP(O))Cl], containing monooxidized BINAP(O) was isolated from a solution of [Rh(BINAP)(µ_2_-Cl)]_2_ and methyl-(*Z*)-α-acetamidocinnamate (MAC) in MeOH, which contained obviously residual traces of oxygen. The molecular structure of [Rh(BINAP(O))Cl] is shown in Fig. 1.

In the title complex, the Rh^I^ atom is η^4^-coordinated to one of the binaphthyl moieties of the partially oxidized BINAP ligand but not to the O atom. Coordination to O atom is instead observed in the complex [Rh(BINAP(O))(CO)Cl] described by Bunten *et al.* (2002). This complex has a square-planar geometry with a CO ligand located *trans* to the O atom. The Rh—P distance of 2.1988 (10) Å in the title complex is by more than 0.1 Å shorter than that in the typical BINAP-rhodium complexes [2.304 (2)–2.335 (2) Å] (Preetz *et al.*, 2010; Tani *et al.*, 1985) and slightly shorter than in [Rh(BINAP(O))(CO)Cl] [2.242 (1) Å]. The Rh—C bond lengths, varying from 2.092 (3) to 2.497 (3) Å, are in the range of the known BINAP-rhodium complexes with benzene (2.226–2.241 Å) (Fischer *et al.*, 2012). The Rh—Cl distance is 2.3222 (15) Å, comparable to that in [Rh(BINAP(O))(CO)Cl] [2.382 (1) Å] (Bunten *et al.*, 2002). An O—H···O hydrogen bond between the methanol solvent molecule and the complex molecule is observed (Table 1).

### Experimental

[Rh(COD)(µ_2_-Cl)]_2_ (0.03 g, 0.06 mmol) and BINAP (0.02 g, 0.03 mmol) were dissolved in tetrahydrofuran at room temperature under anaerobic conditions using standard schlenk-techniques. After stirring for 30 min, the solvent was removed under vacuum. The dark red colored residue was recystallized from CH_2_Cl_2_ to afford [Rh(BINAP)(µ_2_-Cl)]_2_.

[Rh(BINAP)(µ_2_-Cl)]_2_ (0.015 g, 0.01 mmol) and MAC (0.219 g, 1 mmol) were dissolved in 15 ml MeOH and stirred for 6 h under hydrogen. Crystals of the title compound were isolated after two days from the reaction solution, which contained residual traces of oxygen. ^31^P-NMR (300 MHz, THF-d_8_): δ 55.3 p.p.m. (d, J_P—Rh_ = 198,3 Hz), δ 24.4 p.p.m.

### Refinement

H atoms were placed in idealized positions and refined using a riding model, with C—H = 0.93 and 0.98 (CH), 0.96 (CH_3_) and O—H = 0.82 (OH) Å and with *U*_iso_(H) = 1.2(1.5 for methyl and hydroxyl)*U*_eq_(C, O).

### Crystal data

**Table:**

[RhCl(C_44_H_32_OP_2_)]·CH_4_O	*Z* = 1
*M**_r_* = 809.04	*F*(000) = 414
Triclinic, *P*1	*D*_x_ = 1.485 Mg m^−^^3^
Hall symbol: P 1	Mo *K*α radiation, λ = 0.71073 Å
*a* = 9.2108 (18) Å	Cell parameters from 11752 reflections
*b* = 9.7453 (19) Å	θ = 2.0–26.1°
*c* = 11.354 (2) Å	µ = 0.67 mm^−^^1^
α = 103.01 (3)°	*T* = 200 K
β = 104.59 (3)°	Prism, deep-purple
γ = 105.09 (3)°	0.20 × 0.15 × 0.15 mm
*V* = 905.0 (4) Å^3^	

### Data collection

**Table:**

Stoe IPDS-2 diffractometer	6247 independent reflections
Radiation source: sealed X-ray tube, 12 x 0.4 mm long-fine focus	5695 reflections with *I* > 2σ(*I*)
Graphite monochromator	*R*_int_ = 0.025
Detector resolution: 6.67 pixels mm^-1^	θ_max_ = 25.6°, θ_min_ = 2.0°
ω scans	*h* = −11→11
Absorption correction: numerical (*X-SHAPE* and *X-RED*; Stoe & Cie, 2002)	*k* = −11→11
*T*_min_ = 0.787, *T*_max_ = 0.953	*l* = −12→13
10887 measured reflections	

### Refinement

**Table:**

Refinement on *F*^2^	Secondary atom site location: difference Fourier map
Least-squares matrix: full	Hydrogen site location: inferred from neighbouring sites
*R*[*F*^2^ > 2σ(*F*^2^)] = 0.027	H-atom parameters constrained
*wR*(*F*^2^) = 0.043	*w* = 1/[σ^2^(*F*_o_^2^) + (0.006*P*)^2^] where *P* = (*F*_o_^2^ + 2*F*_c_^2^)/3
*S* = 0.96	(Δ/σ)_max_ = 0.001
6247 reflections	Δρ_max_ = 0.64 e Å^−^^3^
462 parameters	Δρ_min_ = −0.34 e Å^−^^3^
3 restraints	Absolute structure: Flack (1983), 2858 Friedel pairs
Primary atom site location: structure-invariant direct methods	Flack parameter: −0.012 (17)

### Special details

**Table:**

Geometry. All e.s.d.'s (except the e.s.d. in the dihedral angle between two l.s. planes) are estimated using the full covariance matrix. The cell e.s.d.'s are taken into account individually in the estimation of e.s.d.'s in distances, angles and torsion angles; correlations between e.s.d.'s in cell parameters are only used when they are defined by crystal symmetry. An approximate (isotropic) treatment of cell e.s.d.'s is used for estimating e.s.d.'s involving l.s. planes.
Refinement. Refinement of *F*^2^ against ALL reflections. The weighted *R*-factor *wR* and goodness of fit *S* are based on *F*^2^, conventional *R*-factors *R* are based on *F*, with *F* set to zero for negative *F*^2^. The threshold expression of *F*^2^ > σ(*F*^2^) is used only for calculating *R*-factors(gt) *etc*. and is not relevant to the choice of reflections for refinement. *R*-factors based on *F*^2^ are statistically about twice as large as those based on *F*, and *R*- factors based on ALL data will be even larger.

### Fractional atomic coordinates and isotropic or equivalent isotropic displacement parameters (Å^2^)

**Table:**

	*x*	*y*	*z*	*U*_iso_*/*U*_eq_	
Rh1	0.25876 (2)	0.03654 (2)	0.95095 (2)	0.01981 (8)	
Cl1	0.46974 (12)	0.12625 (12)	1.14224 (10)	0.0319 (3)	
P1	0.14437 (10)	−0.16409 (9)	0.63082 (8)	0.0219 (2)	
P2	0.32439 (10)	0.24664 (9)	0.90464 (8)	0.0199 (2)	
O1	0.2049 (3)	−0.0336 (2)	0.5902 (2)	0.0281 (6)	
C1	0.1016 (4)	−0.1239 (3)	0.7790 (3)	0.0189 (7)	
C2	0.1186 (4)	−0.2141 (3)	0.8623 (3)	0.0236 (8)	
H2A	0.1757	−0.2850	0.8491	0.028*	
C3	0.0725 (4)	−0.1886 (4)	0.9697 (4)	0.0292 (9)	
H3A	0.1113	−0.2306	1.0371	0.035*	
C4	−0.0324 (4)	−0.1050 (4)	0.9852 (3)	0.0249 (8)	
C5	−0.1039 (4)	−0.1040 (4)	1.0803 (3)	0.0336 (9)	
H5A	−0.0847	−0.1601	1.1351	0.040*	
C6	−0.2012 (5)	−0.0219 (4)	1.0936 (4)	0.0363 (10)	
H6A	−0.2492	−0.0228	1.1564	0.044*	
C7	−0.2288 (5)	0.0639 (4)	1.0122 (4)	0.0367 (10)	
H7A	−0.2960	0.1193	1.0208	0.044*	
C8	−0.1578 (4)	0.0671 (4)	0.9199 (3)	0.0283 (9)	
H8A	−0.1757	0.1260	0.8674	0.034*	
C9	−0.0591 (4)	−0.0169 (3)	0.9041 (3)	0.0207 (7)	
C10	0.0330 (4)	−0.0075 (4)	0.8172 (3)	0.0189 (8)	
C11	0.0256 (4)	0.0962 (3)	0.7374 (3)	0.0164 (7)	
C12	−0.1123 (4)	0.0643 (3)	0.6309 (3)	0.0197 (7)	
C13	−0.2527 (4)	−0.0571 (3)	0.5984 (3)	0.0241 (8)	
H13A	−0.2582	−0.1197	0.6492	0.029*	
C14	−0.3792 (4)	−0.0848 (4)	0.4955 (4)	0.0309 (10)	
H14A	−0.4715	−0.1644	0.4779	0.037*	
C15	−0.3747 (4)	0.0046 (4)	0.4137 (4)	0.0301 (9)	
H15A	−0.4623	−0.0164	0.3419	0.036*	
C16	−0.2399 (4)	0.1223 (4)	0.4416 (3)	0.0258 (8)	
H16A	−0.2358	0.1808	0.3874	0.031*	
C17	−0.1071 (4)	0.1572 (4)	0.5501 (3)	0.0205 (8)	
C18	0.0318 (4)	0.2832 (4)	0.5834 (3)	0.0244 (8)	
H18A	0.0354	0.3449	0.5318	0.029*	
C19	0.1596 (4)	0.3155 (4)	0.6888 (3)	0.0225 (8)	
H19A	0.2481	0.4002	0.7103	0.027*	
C20	0.1577 (4)	0.2200 (3)	0.7659 (3)	0.0191 (8)	
C21	0.2821 (4)	−0.2656 (3)	0.6483 (3)	0.0235 (8)	
C22	0.4175 (4)	−0.2152 (4)	0.7585 (4)	0.0309 (9)	
H22A	0.4307	−0.1352	0.8276	0.037*	
C23	0.5305 (4)	−0.2846 (4)	0.7637 (4)	0.0356 (10)	
H23A	0.6184	−0.2533	0.8374	0.043*	
C24	0.5138 (5)	−0.3997 (4)	0.6605 (4)	0.0390 (10)	
H24A	0.5908	−0.4457	0.6645	0.047*	
C25	0.3842 (5)	−0.4474 (4)	0.5513 (4)	0.0359 (10)	
H25A	0.3744	−0.5243	0.4812	0.043*	
C26	0.2686 (4)	−0.3814 (4)	0.5457 (4)	0.0288 (9)	
H26A	0.1803	−0.4151	0.4719	0.035*	
C27	−0.0417 (4)	−0.2920 (4)	0.5157 (3)	0.0256 (8)	
C28	−0.1017 (5)	−0.2603 (4)	0.4042 (4)	0.0356 (10)	
H28A	−0.0433	−0.1762	0.3897	0.043*	
C29	−0.2464 (5)	−0.3512 (5)	0.3147 (4)	0.0460 (12)	
H29A	−0.2854	−0.3280	0.2405	0.055*	
C30	−0.3323 (6)	−0.4749 (5)	0.3347 (5)	0.0437 (13)	
H30A	−0.4298	−0.5369	0.2739	0.052*	
C31	−0.2753 (4)	−0.5080 (4)	0.4443 (4)	0.0382 (10)	
H31A	−0.3348	−0.5925	0.4577	0.046*	
C32	−0.1311 (6)	−0.4182 (5)	0.5349 (5)	0.0293 (10)	
H32A	−0.0936	−0.4420	0.6090	0.035*	
C33	0.5078 (4)	0.3083 (3)	0.8705 (3)	0.0224 (8)	
C34	0.5148 (4)	0.2566 (4)	0.7479 (3)	0.0264 (8)	
H34A	0.4225	0.1944	0.6812	0.032*	
C35	0.6569 (4)	0.2968 (4)	0.7248 (4)	0.0336 (9)	
H35A	0.6600	0.2618	0.6426	0.040*	
C36	0.7940 (4)	0.3881 (4)	0.8220 (4)	0.0367 (10)	
H36A	0.8892	0.4168	0.8051	0.044*	
C37	0.7910 (4)	0.4376 (4)	0.9452 (4)	0.0393 (10)	
H37A	0.8845	0.4980	1.0115	0.047*	
C38	0.6493 (4)	0.3974 (4)	0.9693 (4)	0.0301 (9)	
H38A	0.6479	0.4301	1.0524	0.036*	
C39	0.3227 (4)	0.4097 (4)	1.0203 (3)	0.0241 (8)	
C40	0.3914 (4)	0.5553 (4)	1.0213 (4)	0.0300 (10)	
H40A	0.4484	0.5721	0.9660	0.036*	
C41	0.3764 (5)	0.6748 (4)	1.1029 (4)	0.0405 (11)	
H41A	0.4242	0.7716	1.1032	0.049*	
C42	0.2902 (5)	0.6505 (5)	1.1843 (4)	0.0441 (11)	
H42A	0.2784	0.7308	1.2388	0.053*	
C43	0.2222 (5)	0.5080 (5)	1.1844 (4)	0.0393 (10)	
H43A	0.1655	0.4920	1.2401	0.047*	
C44	0.2370 (4)	0.3867 (4)	1.1023 (3)	0.0309 (9)	
H44A	0.1892	0.2902	1.1027	0.037*	
O51	0.2263 (4)	0.9663 (3)	0.3525 (3)	0.0569 (9)	
H51	0.2201	0.9544	0.4206	0.085*	
C51	0.2415 (6)	1.1147 (5)	0.3574 (5)	0.0596 (13)	
H51A	0.1561	1.1392	0.3809	0.089*	
H51B	0.2373	1.1259	0.2749	0.089*	
H51C	0.3416	1.1805	0.4198	0.089*	

### Atomic displacement parameters (Å^2^)

**Table:**

	*U*^11^	*U*^22^	*U*^33^	*U*^12^	*U*^13^	*U*^23^
Rh1	0.01928 (16)	0.02279 (17)	0.01809 (17)	0.00796 (13)	0.00489 (13)	0.00812 (14)
Cl1	0.0259 (6)	0.0422 (7)	0.0227 (6)	0.0104 (5)	0.0018 (5)	0.0090 (5)
P1	0.0221 (5)	0.0203 (5)	0.0204 (5)	0.0060 (4)	0.0058 (4)	0.0039 (4)
P2	0.0187 (5)	0.0211 (5)	0.0187 (5)	0.0054 (4)	0.0056 (4)	0.0062 (4)
O1	0.0376 (16)	0.0261 (13)	0.0225 (14)	0.0095 (11)	0.0137 (12)	0.0082 (11)
C1	0.0158 (17)	0.0167 (16)	0.0225 (19)	0.0052 (14)	0.0036 (15)	0.0064 (15)
C2	0.0217 (18)	0.0262 (18)	0.0247 (19)	0.0104 (15)	0.0068 (16)	0.0097 (16)
C3	0.022 (2)	0.035 (2)	0.038 (2)	0.0124 (17)	0.0086 (18)	0.0215 (19)
C4	0.0192 (18)	0.0296 (19)	0.0248 (19)	0.0061 (15)	0.0059 (15)	0.0103 (16)
C5	0.036 (2)	0.041 (2)	0.032 (2)	0.0128 (18)	0.0149 (18)	0.0230 (19)
C6	0.040 (3)	0.050 (3)	0.026 (2)	0.016 (2)	0.020 (2)	0.015 (2)
C7	0.037 (2)	0.040 (2)	0.042 (2)	0.019 (2)	0.021 (2)	0.013 (2)
C8	0.031 (2)	0.031 (2)	0.029 (2)	0.0137 (18)	0.0132 (18)	0.0128 (18)
C9	0.0167 (17)	0.0191 (17)	0.0219 (18)	0.0014 (14)	0.0050 (14)	0.0050 (15)
C10	0.0148 (18)	0.0172 (18)	0.019 (2)	0.0053 (14)	0.0010 (16)	0.0006 (16)
C11	0.0179 (17)	0.0172 (16)	0.0181 (18)	0.0087 (14)	0.0088 (15)	0.0063 (14)
C12	0.0195 (18)	0.0215 (18)	0.0186 (18)	0.0093 (14)	0.0078 (15)	0.0029 (15)
C13	0.025 (2)	0.0230 (18)	0.026 (2)	0.0064 (15)	0.0087 (16)	0.0114 (16)
C14	0.019 (2)	0.031 (2)	0.032 (2)	0.0007 (17)	0.0008 (18)	0.0088 (19)
C15	0.026 (2)	0.034 (2)	0.026 (2)	0.0121 (17)	0.0004 (17)	0.0100 (18)
C16	0.033 (2)	0.029 (2)	0.0194 (19)	0.0134 (18)	0.0079 (16)	0.0117 (16)
C17	0.0237 (19)	0.0197 (18)	0.0209 (19)	0.0119 (15)	0.0084 (15)	0.0052 (15)
C18	0.029 (2)	0.0243 (19)	0.028 (2)	0.0138 (16)	0.0117 (17)	0.0158 (17)
C19	0.0190 (19)	0.0221 (19)	0.026 (2)	0.0045 (15)	0.0079 (16)	0.0085 (16)
C20	0.0203 (19)	0.0171 (17)	0.0202 (19)	0.0087 (14)	0.0081 (15)	0.0018 (15)
C21	0.0221 (19)	0.0210 (18)	0.026 (2)	0.0038 (15)	0.0100 (16)	0.0067 (16)
C22	0.0217 (19)	0.035 (2)	0.034 (2)	0.0069 (16)	0.0095 (17)	0.0089 (18)
C23	0.026 (2)	0.039 (2)	0.046 (3)	0.0122 (18)	0.0128 (19)	0.019 (2)
C24	0.038 (2)	0.035 (2)	0.065 (3)	0.0228 (18)	0.030 (2)	0.028 (2)
C25	0.044 (3)	0.024 (2)	0.046 (2)	0.0133 (18)	0.025 (2)	0.0080 (19)
C26	0.031 (2)	0.027 (2)	0.032 (2)	0.0100 (16)	0.0146 (18)	0.0097 (17)
C27	0.026 (2)	0.0245 (19)	0.025 (2)	0.0111 (16)	0.0074 (16)	0.0036 (16)
C28	0.036 (2)	0.037 (2)	0.027 (2)	0.0082 (19)	0.0062 (19)	0.0086 (19)
C29	0.042 (3)	0.055 (3)	0.027 (2)	0.013 (2)	−0.002 (2)	0.006 (2)
C30	0.031 (3)	0.044 (3)	0.037 (3)	0.005 (2)	0.000 (2)	−0.004 (2)
C31	0.028 (3)	0.028 (2)	0.047 (3)	−0.0001 (18)	0.010 (2)	0.004 (2)
C32	0.026 (2)	0.028 (2)	0.031 (2)	0.0075 (16)	0.0090 (18)	0.0063 (18)
C33	0.0243 (19)	0.0188 (17)	0.0232 (19)	0.0074 (15)	0.0048 (16)	0.0080 (15)
C34	0.023 (2)	0.029 (2)	0.025 (2)	0.0082 (16)	0.0068 (16)	0.0059 (17)
C35	0.038 (2)	0.038 (2)	0.038 (2)	0.0188 (19)	0.022 (2)	0.0191 (19)
C36	0.025 (2)	0.038 (2)	0.063 (3)	0.0137 (18)	0.028 (2)	0.026 (2)
C37	0.021 (2)	0.036 (2)	0.048 (3)	0.0032 (17)	0.0034 (19)	0.007 (2)
C38	0.024 (2)	0.030 (2)	0.031 (2)	0.0036 (17)	0.0105 (17)	0.0061 (17)
C39	0.0214 (18)	0.0278 (19)	0.0184 (18)	0.0089 (15)	0.0014 (15)	0.0032 (15)
C40	0.028 (2)	0.026 (2)	0.031 (2)	0.0091 (17)	0.0036 (17)	0.0072 (19)
C41	0.041 (3)	0.029 (2)	0.039 (3)	0.0150 (19)	−0.002 (2)	0.001 (2)
C42	0.044 (3)	0.045 (3)	0.030 (2)	0.027 (2)	−0.003 (2)	−0.008 (2)
C43	0.041 (2)	0.052 (3)	0.024 (2)	0.023 (2)	0.0105 (19)	0.002 (2)
C44	0.030 (2)	0.033 (2)	0.026 (2)	0.0101 (17)	0.0053 (17)	0.0076 (18)
O51	0.075 (2)	0.058 (2)	0.0428 (19)	0.0185 (17)	0.0309 (19)	0.0154 (16)
C51	0.083 (4)	0.056 (3)	0.054 (3)	0.026 (3)	0.039 (3)	0.022 (3)

### Geometric parameters (Å, º)

**Table:**

Rh1—C1	2.092 (3)	C21—C22	1.408 (4)
Rh1—C10	2.113 (3)	C22—C23	1.377 (5)
Rh1—P2	2.1988 (10)	C22—H22A	0.9300
Rh1—C2	2.289 (3)	C23—C24	1.372 (5)
Rh1—Cl1	2.3222 (15)	C23—H23A	0.9300
Rh1—C3	2.497 (3)	C24—C25	1.372 (5)
P1—O1	1.471 (2)	C24—H24A	0.9300
P1—C27	1.796 (4)	C25—C26	1.376 (5)
P1—C21	1.800 (4)	C25—H25A	0.9300
P1—C1	1.804 (3)	C26—H26A	0.9300
P2—C33	1.809 (4)	C27—C28	1.384 (5)
P2—C20	1.815 (3)	C27—C32	1.386 (5)
P2—C39	1.826 (4)	C28—C29	1.376 (5)
C1—C2	1.437 (4)	C28—H28A	0.9300
C1—C10	1.470 (5)	C29—C30	1.360 (6)
C2—C3	1.381 (5)	C29—H29A	0.9300
C2—H2A	0.9800	C30—C31	1.367 (6)
C3—C4	1.435 (5)	C30—H30A	0.9300
C3—H3A	0.9800	C31—C32	1.374 (6)
C4—C5	1.399 (5)	C31—H31A	0.9300
C4—C9	1.413 (4)	C32—H32A	0.9300
C5—C6	1.362 (5)	C33—C34	1.393 (5)
C5—H5A	0.9300	C33—C38	1.395 (5)
C6—C7	1.399 (5)	C34—C35	1.374 (5)
C6—H6A	0.9300	C34—H34A	0.9300
C7—C8	1.370 (5)	C35—C36	1.369 (5)
C7—H7A	0.9300	C35—H35A	0.9300
C8—C9	1.390 (5)	C36—C37	1.383 (5)
C8—H8A	0.9300	C36—H36A	0.9300
C9—C10	1.457 (5)	C37—C38	1.376 (5)
C10—C11	1.505 (4)	C37—H37A	0.9300
C11—C20	1.379 (4)	C38—H38A	0.9300
C11—C12	1.422 (4)	C39—C44	1.383 (5)
C12—C13	1.405 (4)	C39—C40	1.391 (4)
C12—C17	1.428 (4)	C40—C41	1.377 (5)
C13—C14	1.343 (5)	C40—H40A	0.9300
C13—H13A	0.9300	C41—C42	1.383 (6)
C14—C15	1.410 (5)	C41—H41A	0.9300
C14—H14A	0.9300	C42—C43	1.367 (5)
C15—C16	1.362 (5)	C42—H42A	0.9300
C15—H15A	0.9300	C43—C44	1.392 (5)
C16—C17	1.401 (4)	C43—H43A	0.9300
C16—H16A	0.9300	C44—H44A	0.9300
C17—C18	1.420 (5)	O51—C51	1.403 (5)
C18—C19	1.359 (4)	O51—H51	0.8200
C18—H18A	0.9300	C51—H51A	0.9600
C19—C20	1.413 (4)	C51—H51B	0.9600
C19—H19A	0.9300	C51—H51C	0.9600
C21—C26	1.381 (5)		
			
C1—Rh1—C10	40.93 (13)	C18—C17—C12	118.8 (3)
C1—Rh1—P2	105.25 (9)	C19—C18—C17	121.5 (3)
C10—Rh1—P2	84.52 (10)	C19—C18—H18A	119.2
C1—Rh1—C2	37.95 (11)	C17—C18—H18A	119.2
C10—Rh1—C2	67.61 (12)	C18—C19—C20	119.8 (3)
P2—Rh1—C2	143.03 (8)	C18—C19—H19A	120.1
C1—Rh1—Cl1	155.05 (9)	C20—C19—H19A	120.1
C10—Rh1—Cl1	160.82 (10)	C11—C20—C19	120.8 (3)
P2—Rh1—Cl1	93.61 (5)	C11—C20—P2	115.4 (2)
C2—Rh1—Cl1	120.65 (9)	C19—C20—P2	123.9 (2)
C1—Rh1—C3	63.69 (12)	C26—C21—C22	118.7 (3)
C10—Rh1—C3	73.12 (12)	C26—C21—P1	119.9 (3)
P2—Rh1—C3	155.56 (8)	C22—C21—P1	120.8 (3)
C2—Rh1—C3	33.19 (12)	C23—C22—C21	119.8 (4)
Cl1—Rh1—C3	104.21 (9)	C23—C22—H22A	120.1
O1—P1—C27	111.31 (15)	C21—C22—H22A	120.1
O1—P1—C21	111.22 (15)	C24—C23—C22	120.3 (4)
C27—P1—C21	107.29 (16)	C24—C23—H23A	119.9
O1—P1—C1	115.56 (14)	C22—C23—H23A	119.9
C27—P1—C1	103.97 (16)	C23—C24—C25	120.4 (4)
C21—P1—C1	106.92 (16)	C23—C24—H24A	119.8
C33—P2—C20	109.10 (16)	C25—C24—H24A	119.8
C33—P2—C39	105.40 (16)	C24—C25—C26	120.0 (4)
C20—P2—C39	101.86 (15)	C24—C25—H25A	120.0
C33—P2—Rh1	120.06 (11)	C26—C25—H25A	120.0
C20—P2—Rh1	103.91 (11)	C25—C26—C21	120.8 (4)
C39—P2—Rh1	114.93 (11)	C25—C26—H26A	119.6
C2—C1—C10	115.1 (3)	C21—C26—H26A	119.6
C2—C1—P1	121.6 (3)	C28—C27—C32	118.3 (4)
C10—C1—P1	123.1 (2)	C28—C27—P1	118.4 (3)
C2—C1—Rh1	78.50 (19)	C32—C27—P1	123.3 (3)
C10—C1—Rh1	70.30 (18)	C29—C28—C27	121.0 (4)
P1—C1—Rh1	124.85 (16)	C29—C28—H28A	119.5
C3—C2—C1	120.4 (3)	C27—C28—H28A	119.5
C3—C2—Rh1	81.7 (2)	C30—C29—C28	120.0 (4)
C1—C2—Rh1	63.55 (17)	C30—C29—H29A	120.0
C3—C2—H2A	119.6	C28—C29—H29A	120.0
C1—C2—H2A	119.6	C29—C30—C31	119.9 (4)
Rh1—C2—H2A	119.6	C29—C30—H30A	120.0
C2—C3—C4	122.4 (3)	C31—C30—H30A	120.0
C2—C3—Rh1	65.13 (19)	C30—C31—C32	120.9 (4)
C4—C3—Rh1	88.4 (2)	C30—C31—H31A	119.6
C2—C3—H3A	118.8	C32—C31—H31A	119.6
C4—C3—H3A	118.8	C31—C32—C27	120.0 (5)
Rh1—C3—H3A	118.8	C31—C32—H32A	120.0
C5—C4—C9	119.4 (3)	C27—C32—H32A	120.0
C5—C4—C3	122.2 (3)	C34—C33—C38	118.3 (3)
C9—C4—C3	118.4 (3)	C34—C33—P2	121.0 (3)
C6—C5—C4	120.7 (3)	C38—C33—P2	120.4 (3)
C6—C5—H5A	119.6	C35—C34—C33	120.6 (3)
C4—C5—H5A	119.6	C35—C34—H34A	119.7
C5—C6—C7	119.7 (4)	C33—C34—H34A	119.7
C5—C6—H6A	120.1	C36—C35—C34	120.5 (4)
C7—C6—H6A	120.1	C36—C35—H35A	119.7
C8—C7—C6	120.7 (4)	C34—C35—H35A	119.7
C8—C7—H7A	119.7	C35—C36—C37	120.0 (4)
C6—C7—H7A	119.7	C35—C36—H36A	120.0
C7—C8—C9	120.5 (3)	C37—C36—H36A	120.0
C7—C8—H8A	119.8	C38—C37—C36	119.8 (4)
C9—C8—H8A	119.8	C38—C37—H37A	120.1
C8—C9—C4	119.0 (3)	C36—C37—H37A	120.1
C8—C9—C10	123.5 (3)	C37—C38—C33	120.7 (4)
C4—C9—C10	117.2 (3)	C37—C38—H38A	119.6
C9—C10—C1	121.0 (3)	C33—C38—H38A	119.6
C9—C10—C11	120.9 (3)	C44—C39—C40	118.6 (3)
C1—C10—C11	116.2 (3)	C44—C39—P2	118.5 (3)
C9—C10—Rh1	97.8 (2)	C40—C39—P2	122.7 (3)
C1—C10—Rh1	68.76 (18)	C41—C40—C39	121.1 (4)
C11—C10—Rh1	115.7 (2)	C41—C40—H40A	119.5
C20—C11—C12	120.3 (3)	C39—C40—H40A	119.5
C20—C11—C10	118.8 (3)	C40—C41—C42	119.8 (4)
C12—C11—C10	120.9 (3)	C40—C41—H41A	120.1
C13—C12—C11	123.4 (3)	C42—C41—H41A	120.1
C13—C12—C17	117.9 (3)	C43—C42—C41	119.7 (4)
C11—C12—C17	118.7 (3)	C43—C42—H42A	120.2
C14—C13—C12	121.5 (3)	C41—C42—H42A	120.2
C14—C13—H13A	119.3	C42—C43—C44	120.8 (4)
C12—C13—H13A	119.3	C42—C43—H43A	119.6
C13—C14—C15	121.2 (3)	C44—C43—H43A	119.6
C13—C14—H14A	119.4	C39—C44—C43	120.0 (4)
C15—C14—H14A	119.4	C39—C44—H44A	120.0
C16—C15—C14	118.9 (3)	C43—C44—H44A	120.0
C16—C15—H15A	120.6	C51—O51—H51	109.5
C14—C15—H15A	120.6	O51—C51—H51A	109.5
C15—C16—C17	121.6 (3)	O51—C51—H51B	109.5
C15—C16—H16A	119.2	H51A—C51—H51B	109.5
C17—C16—H16A	119.2	O51—C51—H51C	109.5
C16—C17—C18	122.2 (3)	H51A—C51—H51C	109.5
C16—C17—C12	119.0 (3)	H51B—C51—H51C	109.5
			
C1—Rh1—P2—C33	−97.23 (16)	C3—Rh1—C10—C1	−69.07 (19)
C10—Rh1—P2—C33	−132.86 (16)	C1—Rh1—C10—C11	−109.8 (3)
C2—Rh1—P2—C33	−92.7 (2)	P2—Rh1—C10—C11	11.1 (2)
Cl1—Rh1—P2—C33	66.29 (13)	C2—Rh1—C10—C11	−144.1 (3)
C3—Rh1—P2—C33	−156.5 (3)	Cl1—Rh1—C10—C11	96.4 (4)
C1—Rh1—P2—C20	24.97 (15)	C3—Rh1—C10—C11	−178.9 (3)
C10—Rh1—P2—C20	−10.66 (16)	C9—C10—C11—C20	110.3 (4)
C2—Rh1—P2—C20	29.5 (2)	C1—C10—C11—C20	−85.2 (4)
Cl1—Rh1—P2—C20	−171.52 (12)	Rh1—C10—C11—C20	−7.3 (4)
C3—Rh1—P2—C20	−34.3 (3)	C9—C10—C11—C12	−72.4 (4)
C1—Rh1—P2—C39	135.37 (15)	C1—C10—C11—C12	92.1 (4)
C10—Rh1—P2—C39	99.74 (16)	Rh1—C10—C11—C12	170.0 (2)
C2—Rh1—P2—C39	139.94 (18)	C20—C11—C12—C13	−178.0 (3)
Cl1—Rh1—P2—C39	−61.12 (12)	C10—C11—C12—C13	4.7 (5)
C3—Rh1—P2—C39	76.1 (3)	C20—C11—C12—C17	3.9 (5)
O1—P1—C1—C2	−148.9 (2)	C10—C11—C12—C17	−173.3 (3)
C27—P1—C1—C2	88.9 (3)	C11—C12—C13—C14	−178.7 (4)
C21—P1—C1—C2	−24.5 (3)	C17—C12—C13—C14	−0.7 (5)
O1—P1—C1—C10	37.2 (3)	C12—C13—C14—C15	1.8 (6)
C27—P1—C1—C10	−85.1 (3)	C13—C14—C15—C16	−1.0 (6)
C21—P1—C1—C10	161.6 (3)	C14—C15—C16—C17	−0.8 (5)
O1—P1—C1—Rh1	−50.7 (2)	C15—C16—C17—C18	−177.3 (4)
C27—P1—C1—Rh1	−172.95 (19)	C15—C16—C17—C12	1.9 (5)
C21—P1—C1—Rh1	73.7 (2)	C13—C12—C17—C16	−1.1 (5)
C10—Rh1—C1—C2	−122.2 (3)	C11—C12—C17—C16	177.0 (3)
P2—Rh1—C1—C2	175.53 (17)	C13—C12—C17—C18	178.1 (3)
Cl1—Rh1—C1—C2	37.7 (3)	C11—C12—C17—C18	−3.8 (5)
C3—Rh1—C1—C2	−27.86 (18)	C16—C17—C18—C19	−180.0 (4)
P2—Rh1—C1—C10	−62.26 (19)	C12—C17—C18—C19	0.8 (5)
C2—Rh1—C1—C10	122.2 (3)	C17—C18—C19—C20	2.0 (5)
Cl1—Rh1—C1—C10	159.92 (19)	C12—C11—C20—C19	−1.2 (5)
C3—Rh1—C1—C10	94.4 (2)	C10—C11—C20—C19	176.1 (3)
C10—Rh1—C1—P1	117.2 (3)	C12—C11—C20—P2	179.9 (2)
P2—Rh1—C1—P1	54.9 (2)	C10—C11—C20—P2	−2.8 (4)
C2—Rh1—C1—P1	−120.6 (3)	C18—C19—C20—C11	−1.8 (5)
Cl1—Rh1—C1—P1	−82.9 (3)	C18—C19—C20—P2	177.1 (3)
C3—Rh1—C1—P1	−148.5 (2)	C33—P2—C20—C11	139.8 (3)
C10—C1—C2—C3	−0.2 (4)	C39—P2—C20—C11	−109.1 (3)
P1—C1—C2—C3	−174.6 (3)	Rh1—P2—C20—C11	10.6 (3)
Rh1—C1—C2—C3	61.4 (3)	C33—P2—C20—C19	−39.2 (3)
C10—C1—C2—Rh1	−61.6 (2)	C39—P2—C20—C19	71.9 (3)
P1—C1—C2—Rh1	124.0 (2)	Rh1—P2—C20—C19	−168.3 (3)
C1—Rh1—C2—C3	−130.1 (3)	O1—P1—C21—C26	−91.1 (3)
C10—Rh1—C2—C3	−93.2 (2)	C27—P1—C21—C26	30.9 (3)
P2—Rh1—C2—C3	−137.25 (17)	C1—P1—C21—C26	141.9 (3)
Cl1—Rh1—C2—C3	67.4 (2)	O1—P1—C21—C22	79.4 (3)
C10—Rh1—C2—C1	36.8 (2)	C27—P1—C21—C22	−158.6 (3)
P2—Rh1—C2—C1	−7.2 (3)	C1—P1—C21—C22	−47.6 (3)
Cl1—Rh1—C2—C1	−162.55 (16)	C26—C21—C22—C23	−2.1 (5)
C3—Rh1—C2—C1	130.1 (3)	P1—C21—C22—C23	−172.7 (3)
C1—C2—C3—C4	18.1 (5)	C21—C22—C23—C24	1.9 (5)
Rh1—C2—C3—C4	70.6 (3)	C22—C23—C24—C25	−0.3 (5)
C1—C2—C3—Rh1	−52.6 (3)	C23—C24—C25—C26	−1.1 (6)
C1—Rh1—C3—C2	31.7 (2)	C24—C25—C26—C21	0.9 (5)
C10—Rh1—C3—C2	74.7 (2)	C22—C21—C26—C25	0.7 (5)
P2—Rh1—C3—C2	99.4 (3)	P1—C21—C26—C25	171.4 (3)
Cl1—Rh1—C3—C2	−124.99 (19)	O1—P1—C27—C28	2.9 (4)
C1—Rh1—C3—C4	−95.5 (2)	C21—P1—C27—C28	−119.0 (3)
C10—Rh1—C3—C4	−52.4 (2)	C1—P1—C27—C28	128.0 (3)
P2—Rh1—C3—C4	−27.7 (4)	O1—P1—C27—C32	−174.6 (4)
C2—Rh1—C3—C4	−127.1 (3)	C21—P1—C27—C32	63.5 (4)
Cl1—Rh1—C3—C4	107.9 (2)	C1—P1—C27—C32	−49.6 (4)
C2—C3—C4—C5	167.0 (3)	C32—C27—C28—C29	0.1 (6)
Rh1—C3—C4—C5	−134.1 (3)	P1—C27—C28—C29	−177.6 (4)
C2—C3—C4—C9	−15.3 (5)	C27—C28—C29—C30	−0.4 (7)
Rh1—C3—C4—C9	43.6 (3)	C28—C29—C30—C31	0.5 (8)
C9—C4—C5—C6	1.4 (5)	C29—C30—C31—C32	−0.3 (8)
C3—C4—C5—C6	179.1 (4)	C30—C31—C32—C27	0.0 (8)
C4—C5—C6—C7	−0.7 (6)	C28—C27—C32—C31	0.1 (7)
C5—C6—C7—C8	−0.5 (6)	P1—C27—C32—C31	177.7 (4)
C6—C7—C8—C9	1.1 (6)	C20—P2—C33—C34	−34.8 (3)
C7—C8—C9—C4	−0.4 (5)	C39—P2—C33—C34	−143.5 (3)
C7—C8—C9—C10	−173.5 (4)	Rh1—P2—C33—C34	84.8 (3)
C5—C4—C9—C8	−0.9 (5)	C20—P2—C33—C38	151.5 (3)
C3—C4—C9—C8	−178.6 (3)	C39—P2—C33—C38	42.8 (3)
C5—C4—C9—C10	172.7 (3)	Rh1—P2—C33—C38	−88.9 (3)
C3—C4—C9—C10	−5.0 (4)	C38—C33—C34—C35	−2.1 (5)
C8—C9—C10—C1	−164.0 (3)	P2—C33—C34—C35	−175.9 (3)
C4—C9—C10—C1	22.7 (4)	C33—C34—C35—C36	0.1 (5)
C8—C9—C10—C11	−0.2 (5)	C34—C35—C36—C37	1.6 (5)
C4—C9—C10—C11	−173.5 (3)	C35—C36—C37—C38	−1.3 (6)
C8—C9—C10—Rh1	126.2 (3)	C36—C37—C38—C33	−0.7 (5)
C4—C9—C10—Rh1	−47.1 (3)	C34—C33—C38—C37	2.3 (5)
C2—C1—C10—C9	−20.0 (4)	P2—C33—C38—C37	176.2 (3)
P1—C1—C10—C9	154.3 (2)	C33—P2—C39—C44	−154.2 (3)
Rh1—C1—C10—C9	−86.3 (3)	C20—P2—C39—C44	91.9 (3)
C2—C1—C10—C11	175.5 (3)	Rh1—P2—C39—C44	−19.7 (3)
P1—C1—C10—C11	−10.3 (4)	C33—P2—C39—C40	31.9 (3)
Rh1—C1—C10—C11	109.2 (3)	C20—P2—C39—C40	−82.0 (3)
C2—C1—C10—Rh1	66.3 (2)	Rh1—P2—C39—C40	166.4 (2)
P1—C1—C10—Rh1	−119.4 (2)	C44—C39—C40—C41	0.6 (5)
C1—Rh1—C10—C9	120.3 (3)	P2—C39—C40—C41	174.5 (3)
P2—Rh1—C10—C9	−118.8 (2)	C39—C40—C41—C42	−0.8 (5)
C2—Rh1—C10—C9	86.0 (2)	C40—C41—C42—C43	0.9 (6)
Cl1—Rh1—C10—C9	−33.6 (4)	C41—C42—C43—C44	−0.9 (6)
C3—Rh1—C10—C9	51.2 (2)	C40—C39—C44—C43	−0.6 (5)
P2—Rh1—C10—C1	120.93 (18)	P2—C39—C44—C43	−174.7 (3)
C2—Rh1—C10—C1	−34.25 (17)	C42—C43—C44—C39	0.7 (5)
Cl1—Rh1—C10—C1	−153.8 (2)		

### Hydrogen-bond geometry (Å, º)

**Table:**

*D*—H···*A*	*D*—H	H···*A*	*D*···*A*	*D*—H···*A*
O51—H51···O1^i^	0.82	1.95	2.755 (4)	169

Symmetry code: (i) *x*, *y*+1, *z*.

## References

[bb1] Bunten, K. A., Farrar, D. H., Poe, A. J. & Lough, A. (2002). *Organometallics*, **21**, 3344–3350.

[bb2] Fischer, C., Selle, C., Drexler, H.-J. & Heller, D. (2012). *Z. Anorg. Allg. Chem.* Submitted.

[bb3] Flack, H. D. (1983). *Acta Cryst.* A**39**, 876–881.

[bb4] Preetz, A. (2009). Dissertation, University of Rostock, Germany.

[bb5] Preetz, A., Drexler, H.-J., Schulz, S. & Heller, D. (2010). *Tetrahedron Asymmetry*, **21**, 1226–1231.

[bb6] Sheldrick, G. M. (2008). *Acta Cryst.* A**64**, 112–122. 10.1107/S0108767307043930 18156677

[bb7] Stoe & Cie (2002). *X-AREA*, *X-RED* and *X-SHAPE* Stoe & Cie, Darmstadt, Germany.

[bb8] Tani, K., Yamagata, T., Tatsuno, Y., Yamagata, Y., Tomita, K., Akutagawa, S., Kumobayashi, H. & Otsuka, S. (1985). *Angew. Chem. Int. Ed.* **24**, 217–219.

